# Resources-Stratified Guidelines for Classical Hodgkin Lymphoma

**DOI:** 10.3390/ijerph17051783

**Published:** 2020-03-09

**Authors:** Allan Relecom, Massimo Federico, Joseph M. Connors, Bertrand Coiffier, Irene Biasoli, Alden Moccia, Gilles Salles, Thomas McKee, Raymond Miralbell, Peter Borchmann, John Kuruvilla, Peter Johnson, Franco Cavalli, Martine Delavy, Pierre-Yves Dietrich, Antoine Flahault

**Affiliations:** 1 Department of Oncology, Geneva University Hospitals, Rue Gabrielle-Perret-Gentil 4, 1211 Geneva, Switzerland; Allan.Relecom@gmail.com (A.R.); Pierre-Yves.Dietrich@hcuge.ch (P.-Y.D.); 2 Medical Oncology, CHIMOMO Department, University of Modena and Reggio Emilia, Via Università 4, 41121 Modena, Italy; Massimo.Federico@unimore.it; 3 BC Cancer Agency, Vancouver Centre, 600 West 10th Avenue, Vancouver, BC V5Z 1G1, Canada; jconnors@bccancer.bc.ca; 4 Department of Hematology, Hospices Civils de Lyon, 3 Quai des Celestins, 69002 Lyon, France; flahault.a@wanadoo.fr; 5 Internal Medicine Department, Federal Univsersity of Rio de Janeiro, Av. Pedro Calmon, 550-Cidade Universitária-Rio de Janeiro, RJ 21941-901, Brazil; Irene.Biasoli@gmail.com; 6 Oncology Institute of Southern Switzerland (IOSI) Ospedale Regionale di Locarno “La Carità”, Via Ospedale 1 CH-6600 Locarno; alden.moccia@eoc.ch; 7 Groupe d’étude des lymphomes de l’adulte, CHU de Lyon HCL-GH Sud, 165 Chemin du Grand Revoyet, 69495 Pierre-Benite Cedex, France; Gilles.Salles@chu-lyon.fr; 8 Clinical Pathology Unit, Geneva University Hospitals, Rue Gabrielle-Perret-Gentil 4, 1211 Geneva Switzerland; 9 Faculté de Médecine, Geneva University, Rue Michel-Servet 1, 1206 Genève, Switzerland; 10 Department of Internal Medicine, University Hospital of Cologne, Karpener Str 62, 50924 Cologne, Germany; Peter.Borchmann@uni-koeln.de; 11 Division of Medical Oncology and Hematology, Princess Margaret Cancer Center, Toronto, ON M5G 2C1, Canada; John.Kuruvilla@uhn.ca; 12 Faculty of Medicine, University of Toronto, Toronto, ON M5S 1A8, Canada; 13 Faculty of Medicine, University of Southampton, Southampton S017 1BJ, UK; johnsonp@soton.ac.uk; 14 Institute of Oncology Research (IOR), Via Vela 6, 6500 Bellinzona, Switzerland; Franco.Cavalli@eoc.ch; 15 Institute of Global Health, Faculty of Medicine, University of Geneva, Campus Biotech, Chemin des Mines 9, 1202 Geneva, Switzerland; Martine.Delavy@gmail.com

**Keywords:** Hodgkin lymphoma, resources-stratified guidelines

## Abstract

Hodgkin lymphoma is a haematological malignancy predominantly affecting young adults. Hodgkin lymphoma is a highly curable disease by current treatment standards. Latest treatment guidelines for Hodgkin lymphoma however imply access to diagnostic and treatment modalities that may not be available in settings with restricted healthcare resources. Considerable discrepancies in Hodgkin lymphoma patient survival exist, with poorer outcomes reported in resources-constrained settings. Resources-stratified guidelines for diagnosis, staging and treatment of Hodgkin lymphoma were derived in an effort to optimize patient outcome provided a given setting of available resources. These guidelines were derived based on the framework of the Breast Health Global Initiative stratifying resource levels in basic, core, advanced and maximal categories.

## 1. Introduction

Hodgkin lymphoma (HL) is a lymphoid malignancy with a reported incidence of 79,990 cases worldwide and accounted for 26,167 deaths in 2018 according to the latest GLOBOCAN estimates [1]. It can be classified into either nodular lymphocyte predominant HL or classical HL (cHL), the latter representing 95% of all Hodgkin’s lymphoma. Regional differences in HL prevalence have been reported, with an aged-standardized incidence rate (ASIR) of 3.58 per 100,000 in Western Europe, 1.45 per 100,000 in North Africa and the Middle East, 1.18 per 100,000 in East Asia, down to an estimated 0.4 per 100,000 in Southern Sub-Saharan Africa [2]. Distinct epidemiological patterns have also been described, with a tendency towards younger age at first peak reported in low- and middle-income countries (LMICs) [3]. These geographical discrepancies have been shown to correlate with contrasting rates of EBV positive cases ranging from 90–100% in some parts of Latin America, Africa and Asia, down to 30–50% in Western Europe and North America [4,5]. The factors underlying these differences have not been clearly identified and their therapeutic implications, if any, are not established. Despite its relatively low overall incidence, HL represents a significant burden in the young adult population, where it accounts for 15% of diagnosed malignancies [5].

HL is a highly curable disease by current treatment modalities with a reported 5 years survival of 90% in 2015 in the United States [6]. Reports issued from lower income countries however suggest poorer outcomes in resource-constrained settings [7,8,9,10,11]. Limited availability of diagnostic facilities, imaging modalities and cancer treatment resources, limited access to treatment due to financial, cultural and geographic barriers, and more advanced stage at diagnosis all contribute to this disparity in survival amongst these patient populations [7,8,9].

Whereas limiting long term treatment morbidity and mortality is a key focus of recent and future cHL treatment developments in high income countries today, health systems in many LMICs face a different reality, often struggling to provide their population access to even the most basic and potentially life-saving chemotherapy regimens. Existing evidence-based guidelines outlining current standards in HL diagnosis and treatment fail to take these discrepancies into account, assuming unlimited resources [12,13]. These guidelines are therefore inapplicable in resource-constrained settings, leaving physicians without guidance on how to optimize patient outcome with the limited resources at hand. The concept of resources-stratified guidelines, as originally proposed for breast cancer by the Breast Health Global Initiative (BHGI), was derived to meet this need [14]. Issued through the consensus of a group of experts, resources-stratified guidelines define standards of care according to different levels of available resources. They also allow for incremental improvements in local medical resources for a given cancer, guiding local health system investments to optimize resource allocations. The concept of resource-stratified guidelines has since then been applied to other cancer subtypes, notably by a dedicated National Comprehensive Cancer Network (NCCN) framework. To our knowledge, no resource-stratified guidelines are to date available for cHL. The potential efficacy of existing treatments and the relatively young age of the affected patient population suggest that considerable life-years could be saved by improving treatment of cHL in resource-constrained settings.

Considering the potential benefit expected from optimizing cHL treatment in resource-constrained settings, we aimed at developing a set of evidence-based resources-stratified guidelines for this haematological malignancy.

### Resource Levels

Basic level: Essential services necessary for diagnosis and treatment to be considered. A system lacking any one of the resources defined as basic is unable to provide HL treatment altogether, irrespective of the other resources available.

Core-level: Additional services that provide major improvements in disease outcomes and that are attainable with limited financial means and modest infrastructure.

Enhanced level: Services that provide further moderate improvements in disease outcomes and increase number and quality of therapeutic options available or services that provide major improvements in disease outcomes but are cost prohibitive in lower resource settings.

Maximal level: Resources that may be used in high-resources settings that should be considered lower priority than those in the basic, core and advanced categories on the basis of a modest incremental benefit on outcome and cost and/or impracticability in limited resources environments.

## 2. Materials and Methods

Resource-stratified guidelines for cHL were derived through the consensus of a panel of experts. A survey suggesting options for optimal cHL management according to predetermined levels of resources was designed. Questions addressed cHL diagnosis, staging, first line treatment and treatment at relapse. Definitions of the strata of resources were drawn from those defined by the BHGI in their original publication on breast cancer [14]. The survey was then sent to a selected panel of experts. This panel was composed of an international consortium of medical oncologists, haematologists, radiation-oncologists and pathologists, selected based on their expertise in Hodgkin’s lymphoma. A modified Delphi approach was used to derive the panel’s consensus on the resources-stratified treatment propositions outlined in the questionnaire. A first draft of the manuscript was issued from the collected information. This manuscript was then submitted to participating experts for final approval and validation as second and final round. An enlarged panel of experts was solicited for this final validation to ensure a broad endorsement of our propositions. Our resources-stratified recommendations for cHL diagnosis and staging are outlined in Section 3.1 of the text and summarized in Table 1. Recommendations for cHL treatment are discussed in Section 3.2 of the text and presented in Table 2.

### Search Strategy and Selection Criteria

References for this review were identified through searches of pubmed with the search terms “Hodgkin lymphoma”, “diagnosis”, “staging”, “chemotherapy”, “radiotherapy”, “limited disease”, “advanced disease”, “relapsed” and “refractory” from 1970 to December 2019. We selected randomized controlled trials as well as the latest guidelines of the National Comprehensive Cancer Network and of the European Society for Medical Oncology. Incidence and mortality data were searched from GLOBOCAN 2018 and individual country registries.

## 3. Results

### 3.1. Diagnosis, Pathology and Staging of cHL

- Diagnostic Biopsy

Diagnosis of HL should be based on the histopathological evaluation of an excisional lymph-node biopsy or surgical tissue sample. This should be preferred to large core needle biopsies which remain an acceptable alternative. Fine needle aspirations do not allow for sufficient material nor adequate analysis of the architecture of the tumor to be made for a reliable diagnosis and are not considered appropriate, regardless of the resource setting.

- Pathology

Hodgkin lymphoma is defined by a characteristic histopathologic picture composed of a minority of disease-defining Reed-Sternberg cells associated with other Hodgkin cells and surrounded by a heterogenous inflammatory infiltrate. The appearance of the neoplastic cells as well as the composition of the inflammatory background and its architecture distinguishes the four subtypes of HL. A subset of cases presenting a highly characteristic histopathologic aspect may allow for a reasonably reliable diagnosis to be made based on light microscopy alone if read by an experienced pathologist. Immunophenotyping of the neoplastic cells allows for diagnostic confirmation. It is also a valuable tool in distinguishing cHL from nodular lymphocyte predominant HL and non-Hodgkin lymphoma (NHL) subtypes. Immunohistochemistry (IHC) testing for expression of CD (cluster differentiation) 15, CD30, pan-B markers CD20 and CD79a as well as a pan-T marker CD3 is considered sufficient for the diagnosis of a majority of HL. These IHC tests are fundamental to identify cHL cells (expressing CD15 and CD30 and are CD45 negative in a vast majority of cases, with variable positivity for CD20) from the nodular lymphocyte predominant subtype (staining positively for CD20 and CD45 whilst CD15 and CD30 negative). These also serve to distinguish HL from NHL variants (such as variants of diffuse large B cell lymphoma, high grade B cell lymphoma and anaplastic lymphoma) where additional IHC testing may prove necessary in rare situations. As such, although diagnosis made on light microscopy alone may be appropriate in selected cases, complementary IHC testing should be mandatory if available, to allow for diagnostic confirmation. We therefore consider light microscopy with haematoxylin-eosin coloring a basic resource for HL diagnosis. IHC is considered a core resource considering the considerable contribution of IHC for diagnostic confirmation and workup of more complex cases. Further IHC, to clarify rare cases in which CD30 and CD15 are negative would be considered appropriate for settings with advanced resources.

- Staging and Risk Group Stratification

Existing guidelines for HL propose distinct treatment algorithms based on risk group stratification, classifying disease as limited, intermediate and advanced, or early favorable, early unfavorable and advanced according to the European Organization for Research and Treatment of Cancer/Lymphoma Study Association (EORTC/LYSA), the German Hodgkin Study Group (GHSG) and the NCCN respectively [12,13]. These risk groups are defined by radiologic staging according to the Ann Arbor classification as well as on clinical and biological factors. The application of these treatment algorithms therefore implies access to computed tomography for staging or combined positron emission and computed tomography (PET-CT), if available. Although increasingly available, access to computed tomography remains costly and limited in remote regions of LMIC [15]. Classic HL is a highly curable disease with chemotherapy alone irrespective of stage at diagnosis. Treatment of cHL should therefore not be withheld on grounds of lack of availability of adequate radiologic equipment for staging. As such, CT scan is considered a core resource for HL staging. When CT scan is not available, staging should be based on a chest X-ray and abdominal ultrasound if available. PET-CT is regarded as the radiologic workup of choice if available according to the Lugano Classification [16]. PET-CT has also proven to be a very valuable tool in cHL management when repeated after initiation of therapy, providing prognostic and predictive information leading to potential treatment modulation. Its prohibitive cost, its limited availability and practicality in low resources settings however classify it as an enhanced resource. Bone marrow biopsy is not recommended if PET-CT is available provided its high sensitivity for detection of bone marrow involvement. If PET-CT is not available, indication for bone marrow biopsy should be restricted to CT-scan stage I/II patients presenting with B symptoms and/or elevated sedimentation rate to confirm non-advanced disease.

- Laboratory Tests and Organ Function Testing

All patients considered for chemotherapy treatment should be offered a full blood count and a chemistry panel testing for kidney function and liver enzymes and a sedimentation rate. The latter laboratory tests are considered basic resources for cHL treatment. Serology testing for HIV, hepatitis B and C are strongly encouraged.

An anthracycline and bleomycin are present in standard chemotherapy regimens for cHL treatment. These are characterized by potential cardiac and pulmonary toxicity respectively. Cardiac testing by echocardiography and lung function testing should be offered as part of the pre-treatment workup. The panel, however, considers that the unavailability of these tests should not lead to the withholding of chemotherapy treatment if a thorough medical history and physical examination do not suggest an underlying heart or pulmonary condition. Echocardiography and lung function testing are therefore considered core resources for cHL diagnostic workup. Reproductive counseling should be offered to patients before chemotherapy treatment initiation if the cost and level of available resources allows. Of note, the infertility rate associated with the classic HL chemotherapy backbone of ABVD (doxorubicin, bleomycin, vinblastine and dacarbazine) is reported to be less than 10%, but may reach 20% in women > 35 years old treated with the same regimen [17,18].

### 3.2. Treatment of cHL

#### 3.2.1. Treatment Modalities

- Chemotherapy and Immunotherapy

Strong disparities in access to chemotherapy drugs are reported worldwide, with significant proportions of agents listed as essential medicines by the World Health Organization (WHO) being unavailable in many LMICs today [19]. The high cost of the more recent medications having been granted Food and Drug Administration (FDA) approval for cHL treatment suggest this gap may further widen in the years to come. ABVD however remains to date the standard chemotherapy backbone of cHL treatment showing superior outcomes in limited and advanced disease as compared to the prior reference MOPP (mechlorethamine, vincristine, procarbazine and prednisone) regimen [20] with markedly reduced short-term and long-term toxicity [21]. ABVD chemotherapy is widely available and is included in WHO’s Essential Medicines list. The reported toxicity profile of this regimen issued from studies carried out in high resources settings is acceptable with rates of grade 3–4 neutropenia of 30–34%, low rates of severe infections (2%) and treatment related mortality [22]. Of note, comparably higher rates of treatment related mortality, in part due to high rates of severe infectious complications amongst lower socio-economic groups, have been reported with ABVD chemotherapy in low resource settings [10]. This regimen can be safely administered without the need of neutrophil growth factor support [23], with the exception of patients >60 years old and of vulnerable populations in low resources settings where this should be more closely considered. Bleomycin induced lung toxicity (BLT) has an incidence ranging from 15% to 53% with the ABVD regimen with an associated mortality of 4% to 5% [24,25,26]. Older age, pulmonary irradiation, altered renal function, Granulocyte-colony stimulating factor (G-CSF) support and tobacco history have been described as possible risk factors for BLT [27,28]. Omission of bleomycin has been associated with loss of efficacy of the ABVD regimen [29]. Careful weighing of potential risks and benefits of bleomycin administration to patients presenting with such risk factors is warranted in contexts where access to baseline and follow-up pulmonary function testing is restricted. Of note, high rates of bleomycin induced lung toxicity have been reported in patients older than 60 years of age treated with more than two cycles of ABVD [30]. Based on this observation, we recommend omission of bleomycin after the second cycle of ABVD chemotherapy in patients >60 years old. Taking this into account, the ABVD chemotherapy is an essential resource for cHL management with a favorable safety profile adapted to resource-constrained settings. It is considered a basic resource for cHL management.

The escalated BEACOPP (bleomycin, etoposide, doxorubicin, cyclophosphamide, vincristine, procarbazine, prednisone) regimen developed by the German Hodgkin Study Group (GHSG) is more effective than ABVD in terms of tumor control with consistent improvements in progression-free survival (PFS) when compared with ABVD, correlating with a trend towards increased overall survival (OS) that did not reach statistical significance in direct head to head comparisons [22,31,32,33]. A network meta-analysis comparing upfront ABVD with escalated BEACOPP has shown a statistically significant 10% advantage in OS at 5 years in favor of the escalated BEACOPP regimen [34]. Haematologic toxicity with this more intensive chemotherapy is however considerable with grade 3–4 leucopenia reported in 98% of patients correlating with 22% of grade 3–4 infections [35]. Experienced physicians, adequate supportive care, close monitoring and access to G-CSF support are mandatory for safe administration of the escalated BEACOPP regimen. As such, it is impracticable in many resource-constrained settings and is considered an enhanced resource for cHL treatment.

The antibody-drug conjugate brentuximab vedotin is a highly potent agent in relapsed/refractory cHL and was also tested in first line combined with an AVD (doxorubicine, vinblastine and dacarbazine) chemotherapy backbone [36]. PD-1 blockade by the immune checkpoint inhibitors nivolumab and pembrolizumab has also shown considerable activity in the relapsed/refractory setting. Both agents are under investigation in first line treatment regimen combinations in ongoing trials, with to date no available data to base recommendations for their use in these indications. Cost and availability are also limiting factors for the use of these drugs resource-constrained settings. Taking this into account, these agents are classified as enhanced resources in cHL management.

- Radiotherapy

Classic Hodgkin lymphoma is a highly radio-sensitive disease. Access to radiotherapy is often restricted in low-resource settings, with only an estimated 40% to 50% of worldwide radiotherapy needs being met today. This restricted availability of radiotherapy is notably pronounced in Africa where 29 out of 54 countries are devoid of a functioning radiotherapy unit [37]. In LMICs with functioning radiotherapy units, access to radiotherapy is often limited by high demands, long delays and treatment costs [38]. Enhancing access to radiotherapy is critical in developing a comprehensive HL management program as it allows for a significant broadening of treatment alternatives and a prognostic advantage in the limited disease setting. Radiotherapy may also be a valuable treatment modality in given advanced disease indications as well as in palliation. Taking notice of the restricted availability of radiotherapy in many LMICs and the curative potential of chemotherapy in both limited and advanced stage disease, the panel placed an emphasis on chemotherapy only options in restricted resources settings. The incremental gain in disease control and/or survival expected from additional or complementary radiotherapy was nonetheless highlighted in indications where such an advantage had been clearly demonstrated. Radiotherapy was classified by the panel as an enhanced resource for cHL management.

#### 3.2.2. Treatment Algorithms

- Early Stage Hodgkin’s Lymphoma

A combined modality treatment approach is considered the current standard of care in resource-unlimited settings for early and intermediate stage disease. These consist in a limited number of chemotherapy cycles followed by radiotherapy [12,13]. These recommendations are based on a number of studies suggesting modestly inferior disease control in limited stage cHL treated with chemotherapy only as opposed to combined modality treatment (CMT) [39,40]. Meyer et al. showed that patients with stage I to IIA non-bulky cHL treated with four to six cycles of ABVD chemotherapy had inferior disease-free survival (DFS) as compared to combined modality treatment groups [41]. A meta-analysis of five randomized controlled studies comparing chemotherapy alone versus CMT for early stage cHL confirmed a modest PFS and OS advantage for CMT [42].

CMT, which implies an access to a reliable staging imagery (CT scan, PET-CT if available) to confirm localized disease and to a functioning accessible radiotherapy unit, is recommended when these resources are available. Two to three cycles of ABVD followed by 20 Gy involved field radiotherapy (IFRT) and four cycles of ABVD followed by 30 Gy are considered standard of care for limited and intermediate stage disease, respectively [43]. A treatment alternative of two cycles of ABVD followed by two cycles of escalated BEACOPP and 30 Gy IFRT has been shown to provide a modest freedom from treatment failure (FFTF) and PFS advantage over four cycles of ABVD followed by equivalent radiotherapy in intermediate stage patients, at the cost of increased acute toxicity with no benefit in OS. This latter treatment protocol should only be considered in enhanced resources settings.

Early stage disease at presentation is, however, seldom seen in lower resources settings, with 6% of early favorable and 65% of advanced disease at diagnosis recently reported in a Brazilian registry [7]. In settings where CMT cannot be proposed due to lack of the necessary staging or treatment resources, our panel considers a chemotherapy only treatment a standard of care. Long term follow-up from a number of studies show that ABVD chemotherapy only is a curable treatment for a majority of patients presenting with early and intermediate stage disease [41,44]. In this setting, a standardized treatment of six cycles of ABVD chemotherapy is recommended, with reported 5 years FFP (freedom from progression) and OS of 81% and 90% respectively, in early stage disease [44]. Longer follow-up from ABVD chemotherapy only arms administered to patients with stage I/IIA non-bulky disease, reporting rates of 12 years PFS and OS of 87% and 94% respectively, further support the long-term disease control this treatment option may offer [41]. Attempts at limiting chemotherapy to four cycles of ABVD in early stage disease when radiotherapy is omitted results in slightly higher rates of early relapse [40].

A number of studies incorporated interim PET-CT-based treatment intensity adaptation in an attempt to exploit the predictive and prognostic information it provides. Omitting radiotherapy in early and intermediate risk group early-PET-CT (ePET) negative patients resulted in modestly higher rates of relapse and did not show non-inferiority with regards to PFS when compared to CMT [43,45]. The results of these studies along with the outcomes reported by the CALGB group in a phase II study nonetheless show that three to four cycles of ABVD with no addition of radiotherapy provides excellent outcomes for a vast majority of patients with stage I/II presenting favorable prognostic features and a negative ePET, with reported 3 and 5 years PFS of 91% and 87% respectively [46]. Treatment intensification with escalated BEACOPP + INRT (involved nodal radiotherapy) in early and intermediate-risk patients presenting with a positive PET-CT after two cycles of ABVD resulted in improved PFS when compared with pursuit of ABVD + INRT [40], with a trend towards improved OS which was not statistically significant. Such treatment algorithms, which imply access to repeated PET-CT imaging, are now considered standard of care in many centers of expertise. Interim-PET-CT based treatment intensity modulation protocols are classified as a maximal resource, provided their impracticability in low resource settings.

- Advanced Disease

Advanced cHL disease treated with six to eight cycles of ABVD chemotherapy was shown to result in 5 years FFS and OS of 61% and 73%, respectively [20]. This chemotherapy regimen is considered a standard of care for advanced cHL in the basic and core resource setting. Four to six cycles of escalated BEACOPP can be considered as an alternative regimen, offering a potential benefit in tumor control over ABVD at the cost of increased short- and long-term toxicity [22,47]. Escalated BEACOPP chemotherapy is not recommended for patients above 60 years old due to excessive toxicity in this patient population [47]. Extensive experience and supportive care resources are warranted to conduct escalated BEACOPP chemotherapy, which is therefore classified as an option to be considered in the enhanced resource setting for advanced cHL. Brentuximab vedotin in combination with AVD chemotherapy has recently shown a gain of 4.9 percentage points (HR = 0.77, *p* = 0.04) in two-year-modified PFS over ABVD, at the cost of increased toxicity, with—to date—still immature data on overall survival [36]. This regimen should only be considered as a treatment alternative in the maximal resource setting.

When PET-CT is available, an interim PET-CT is recommended after two cycles of frontline chemotherapy, providing validated prognostic information [48]. A number of prospective randomized studies investigating treatment intensification, with BEACOPP escalated chemotherapy or high dose chemotherapy followed by autologous stem cell transplant (ASCT), in patients treated with a prior two cycles of ABVD chemotherapy presenting a positive interim PET-CT, showed higher PFS as compared to historical controls [49]. These treatment strategies are only relevant for the 15 percent of patients with advanced cHL who have a positive interim PET-CT. Longer follow-up of these results are needed before they can be considered for formal treatment recommendations. Treatment de-escalation strategies for patients reaching ePET negativity have also shown favorable toxicity and safety outcomes. Bleomycin can be omitted in ePET-negative patients after two cycles of ABVD chemotherapy thereby reducing rates of treatment related pulmonary toxicity, without significantly impacting treatment outcome [50]. Patients treated frontline with two cycles of escalated BEACOPP who become PET negative can also be safely treated to a total of only four cycles of escalated BEACOPP chemotherapy or four additional cycles of ABVD without impacting on the treatment outcome [51,52]. Such treatment approaches should only be considered in the maximal resource setting.

Radiotherapy has limited and debated indications in advanced cHL. Studies from the GELA (groupe d’etude des lymphomes de l’adulte) [53], EORTC groups and a meta-analysis have suggested no benefit of consolidation radiotherapy after a full course of chemotherapy in advanced cHL with results even suggesting a potential detrimental effect in this indication [54,55]. This contradicts results from a study conducted at the Tata Memorial Hospital suggesting an improved PFS and OS in favor of consolidation radiotherapy administered to patients with advanced disease in complete remission after six cycles of ABVD [56]. Improved outcome from additional radiotherapy in advanced cHL when administered to patients with initial bulky disease and residual disease after a full course of chemotherapy is also suggested by the results from the UKLG LY09 study [57]. Additional radiotherapy, when available, can be considered in the context of an initial bulk and/or residual disease after the full course of chemotherapy. Radiotherapy can however be safely omitted for the patients with PET negative <2.5 cm residual disease treated with an escalated BEACOPP chemotherapy regimen [51,58]. The modest added value of radiotherapy in these restricted indications of advanced cHL limit its relevance in a resource-constrained setting.

- Relapsed and Refractory Disease

Treatment failure is reported in 10% of patients treated for early stage cHL and 30% to 40% of those with advanced cHL disease will either be shown to be primarily refractory to frontline therapy or relapse after the latter [59,60]. Retrospective and prospective randomized studies set high dose salvage chemotherapy followed by ASCT as the treatment standard for relapsed or primary refractory cHL allowing for 5 years overall survival rates ranging from 35% to 50% [61,62,63,64]. The treatment costs, medical equipment, expertise and the supportive care resources mandatory for high dose chemotherapy and ASCT limit its accessibility in most areas of low- and middle-income countries. Referral to competent treatment centers should be encouraged when feasible.

Brentuximab vedotin has shown considerable activity in a patient population progressing after ASCT or two prior chemotherapy regimens in a pivotal phase II trial, showing an impressive overall response rate (ORR) of 75% and a median OS of 22.4 months. A good proportion of patients having achieved a CR remained disease free at a median of 53 months, 12 out of 28 patients with no further treatments [65]. Brentuximab vedotin has also shown a PFS advantage when given as a maintenance for up to 16 cycles as compared to placebo in a randomized controlled phase III study in high risk patients after ASCT [66]. Based on these results, Brentuximab vedotin is recommended for patients progressing after ASCT or for patients unfit for ASCT progressing after at least two prior chemotherapy regimens. Considering the limited availability of this drug in resources-constrained settings but its considerable benefit, Brentuximab vedotin is classified as an enhanced resource for the management of relapsed/refractory cHL.

In the enhanced and maximal resource setting, patients relapsing after ASCT who have received Brentuximab vedotin may be considered for a number of treatment options. These include allogenic stem cell transplantation and immune-check point inhibitors. Retrospective and prospective studies support allogenic stem cell transplantation as a potentially curative treatment option which should be restricted to young patients with few comorbidities and chemo-sensitive disease [67,68]. PD-1 blockade with nivolumab or pembrolizumab has shown considerable activity in this setting in relapsed/refractory patients progressing after ASCT. Phase II studies with both agents showed overall response rates of close to 70% and CRs of roughly 20% in this highly pre-treated population with long term PFS ranging from 11 to 18 months [69,70]. Immune check-point inhibitors are considered an enhanced resource (significant benefit but limited availability in low resources settings) for patients progressing after ASCT and/or after prior brentuximab vedotin treatment. Of note, contrary to allogenic stem cell transplantation which can only be considered in highly equipped centers of expertise, immune-check point inhibitors are drugs that do not require complex supportive care resources to be prescribed safely. Improving accessibility of these drugs by addressing the issue of their cost in lower resources-settings is warranted to provide relapsing or refractory cHL patients with a feasible treatment option.

The remaining treatment options for patients presenting with relapsed/refractory cHL are of more limited benefit. Some data, however, suggests potential long-term disease control with standard dose chemotherapy regimens at relapse. Reports of retreatment with a MOPP-ABVD regimen at relapse revealed 8-year freedom from second progression and OS of 52% and 63%, respectively, in a subgroup of patients having achieved a first remission of more than 12 months [71]. A standard dose chemotherapy regimen re-challenge can be considered in basic and core resources settings when access to the more potent treatment modalities described above is limited and when time to first treatment failure exceeds 12 months, with care to limit the total anthracyclin dose under cardiotoxic limits. Gemcitabine and bendamustine have been studied in relapsed and refractory cHL patients with an overall response rate of 39% and 56% with median duration of response of 6.7 and 5 months respectively [72,73]. Single agent vinblastine has also been studied in this setting providing comparable outcomes, with an overall response rate of 59% and a median event-free survival of 8.3 months [74]. The short-term and relative benefit of these chemotherapy regimens classifies these resources as enhanced resources for heavily pretreated relapsed/refractory cHL.

Salvage radiotherapy for localized relapsed/refractory cHL has been reported to provide 23% to 44% long-term disease control. Advanced disease at relapse and short duration of first remission are considered poor outcome predictors when radiotherapy is proposed in this setting [75,76]. When available, radiotherapy can be considered as a valuable tool offering a chance of long-term benefit, when extension of disease at relapse enables it and when other treatment modalities used in this context (such as ASCT) are unavailable.

## 4. Conclusions

Hodgkin’s lymphoma is a lymphoid malignancy affecting a predominantly young patient population with very high cure rates when adequate treatment is offered. Considerable life-years can be expected to be saved by optimizing cHL treatment in settings where resources are limited. This set of resources-stratified guidelines identifies the fundamental basic resources required to treat this disease and outlines those that provide the most additional benefit. The main basic resources identified for cHL treatment are relatively simple and accessible in many resource-restricted settings. This implies that basic treatment for cHL can and should be made available, even when health system resources are scarce. This contrasts with a majority of resources that provide significant additional improvements in cHL patient outcome which are unfortunately scarcely accessible due to high cost, unavailability, or lack of the necessary medical infrastructure or expertise. Incremental improvements in health systems guided by selecting resources according to their additional benefits will optimize impact on a public health level. Investment in implementation research is of paramount importance to gain insight into the benefits and risks of cHL treatments in health systems with limited resources.

## Figures and Tables

**Table 1:** Diagnosis and staging recommendations according to level of available resources.

Diagnostic Modality	Level of Available Resources			
	Basic	Core	Advanced	Maximal
Laboratory tests and Imaging	- Full blood count, sedimentation rate - Basic chemistry with kidney function and liver enzymes - Serologic screening for HIV/Hepatitis B and C strongly encouraged - Chest X-Ray and abdominal US if available	- Serologic testing for HIV, Hepatitis B and C - CT scan - Bone marrow biopsy *	- PET-CT	- Interim PET-CT
Pathology	- Excisional biopsy preferred, core needle biopsy as an alternative - Light microscopy with basic HE staining - Basic complementary IHC strongly encouraged	- Basic IHC testing for CD15, CD30, pan B makers CD20 and CD79a and panT marker CD3	Full IHC testing (core + IRF4/MUM1 and PAX5). Further immunohistochemistry depending on results.	
Complementary workup	- Full medical history and clinical examination (with a focus on pre-existing heart and pulmonary conditions)	- Cardiac ultrasonography - Pulmonary function testing	- Reproductive counseling when appropriate	

* Indication for bone marrow biopsy is limited to CT-scan based stage I/II disease with either B symptoms or elevated sedimentation rate to confirm non-advanced disease, in contexts where radiotherapy is available for combined modality treatment (CMT) to be considered, and when positron emission tomography-computed tomography (PET-CT) cannot be proposed. HE—Hematoxilin Eosin; IHC—immunohistochemistry; CD—cluster differentiation.

**Table 2:** Treatment recommendations according to available level of resources.

Disease Stage	Level of Available Resources			
	Basic	Core	Enhanced	Maximal
Undetermined Stage	Six Cycles of ABVD Chemotderapy	-	-	-
Limited stage	-	Six cycles of ABVD or CMT * with 2–4 cycles of ABVD chemotherapy and 20–30 Gy IFRT/ISRT (number of ABVD chemotherapy cycles and radiotherapy dose depending on risk group)	CMT as defined in the core level setting - two cycles of ABVD followed by two cycles of escalated BEACOPP ** and 30 Gy IFRT/ISRT as a possible alternative for intermediate stage	Two cycles of ABVD with interim PET-CT, intensification with two cycles of escalated BEACOPP ** + 30 Gy ISRT if interim PET positive, one additional cycle of ABVD + 20 Gy ISRT if interim PET negative
Advanced	6 cycles of ABVD chemotherapy	6 cycles of ABVD chemotherapy	- 6 cycles of escalated BEACOPP ** as an alternative to 6 to 8 cycles of ABVD chemotherapy - Complementary radiotherapy for initial bulky or residual disease or >2.5cm PET + residual disease (if treated with escalated BEACOPP **)	2 cycles of ABVD chemotherapy and interim PET-CT - If PET positive, consider treatment intensification - 2 cycles of escalated BEACOPP ** as an alternative initial therapy, if PET negative, restrict chemotherapy to 2 additional escalated BEACOPP ** or 4 cycles of ABVD, complementary radiotherapy as outlined in the enhanced level setting
Relapsed/Refractory disease	Referral to primary care center for ASCT if possible	Referral to primary care center for ASCT if possible - Palliative single agent gemcitabine, bendamustine or vinblastine chemotherapy	High dose chemotherapy followed by ASCT if age/comorbidities permits - Brentuximab vedotin as consolidation after ASCT, if relapse after ASCT or if progression after 2 prior lines of chemotherapy - PD-1 inhibitors nivolumab or pembrolizumab if progression after ASCT and/or prior brentuximab vedotin	Allogeneic SCT if progression after ASCT to be considered

* Combined Modality Treatment, to be preferred to chemotherapy alone in limited stage disease when appropriate staging resources and radiotherapy are available. ** Escalated BEACOPP should not be given to patients over the age of 60 years. CMT—combined modality treatment; IFRT—involved field radiotherapy/ISRT—involved site radiotherapy; ASCT—autologous stem cell transplantation; PD—progressive disease.
